# Supplementary material for: Preclinical efficacy and safety analysis of gamma-irradiated inactivated SARS-CoV-2 vaccine candidates
Source: Sci Rep. 2021 Mar 11;11:5804. doi: 10.1038/s41598-021-83930-6 (PMC7970959; doi:10.1038/s41598-021-83930-6)
Supplement: Supplementary file 1 — Supplementary Information 1. [file 41598_2021_83930_MOESM1_ESM.pdf]

Preclinical efficacy and safety analysis of gamma-irradiated inactivated SARS-CoV-2 vaccine candidates

Gozde Sir Karakus, Cihan Tastan, Derya Dilek Kancagi, Bulut Yurtsever, Gamze Tumentemur, Sevda Demir, Raife Dilek Turan, Selen Abanuz, Didem Cakirsoy, Utku Seyis, Samed Ozer, Omer Elibol, Muhammer Elek, Gurcan Ertop, Serap Arbak, Merve Acikel Elmas, Cansu Hemsinlioglu, Ayse Sesin Kocagoz, Ozden Hatirnaz Ng, Sezer Akyoney, Ilayda Sahin, Ugur Ozbek, Dilek Telci, Fikrettin Sahin, Koray Yalcin, Siret Ratip, Ercument Ovali

supplementary figure

Supplementary Figure 1

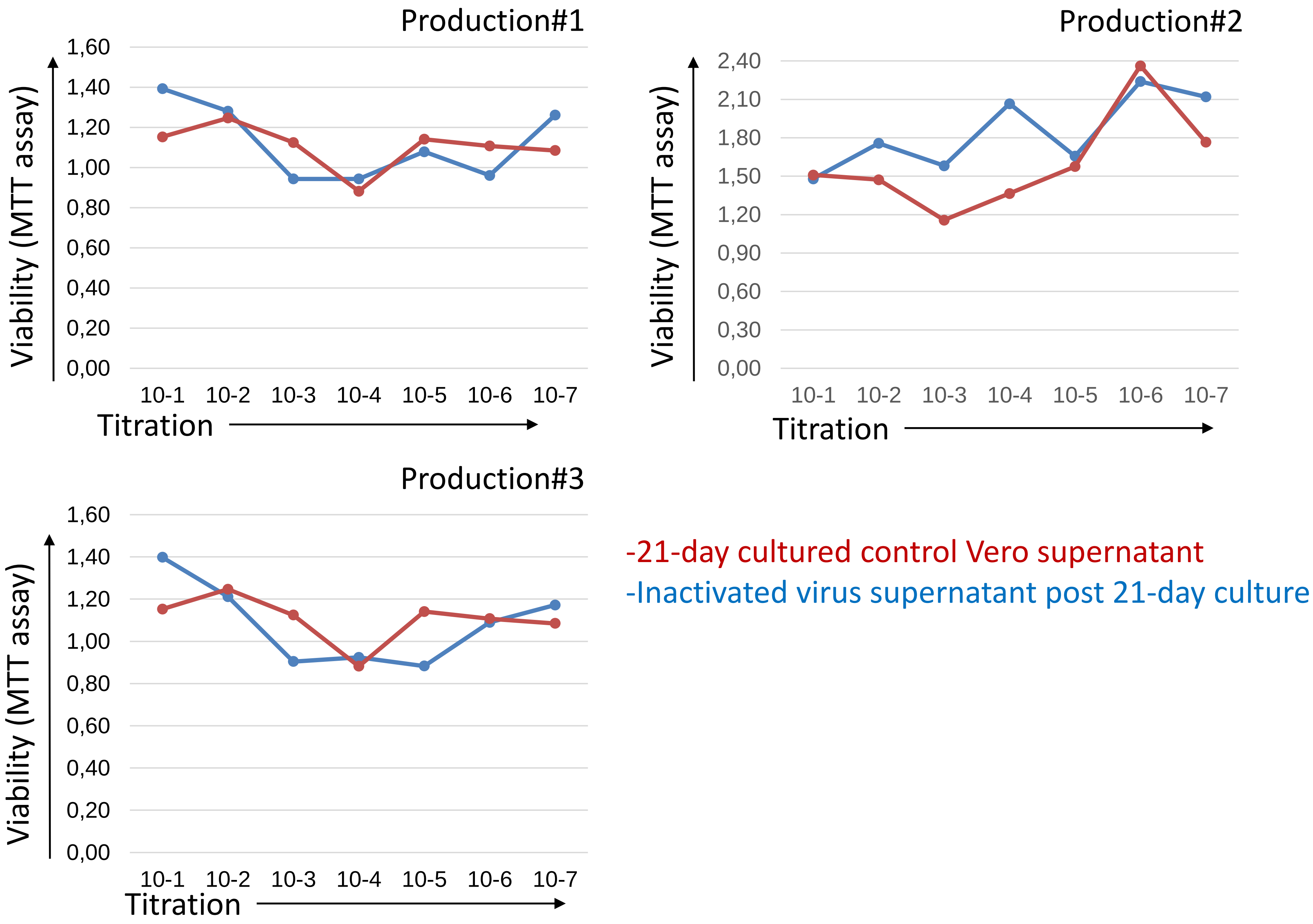

Supplementary Figure 1: Colorimetric MTT viral culture assay. Three independent inactivated virus production was tested for the inactivation by inoculating Vero cells for 21-day of culturing with subsequent passages using 1:2 refreshing culturing media. At the end of the 21-day culturing, the active virus was expected to be propagated if existed in the vaccine samples. To assess the cytopathic effect of the potential infective virus, an MTT assay was performed as described 14. The Redline is to control Vero supernatant post 21-day culturing. The blue line is the supernatant of Vero cells inoculated with inactivated Virus vaccine sample post 21-day culturing.
